# Marine-Derived Biocatalysts: Importance, Accessing, and Application in Aromatic Pollutant Bioremediation

**DOI:** 10.3389/fmicb.2017.00265

**Published:** 2017-02-20

**Authors:** Efstratios Nikolaivits, Maria Dimarogona, Nikolas Fokialakis, Evangelos Topakas

**Affiliations:** ^1^Industrial Biotechnology & Biocatalysis Group, Biotechnology Laboratory, School of Chemical Engineering, National Technical University of AthensAthens, Greece; ^2^Division of Pharmacognosy and Chemistry of Natural Products, Department of Pharmacy, University of AthensAthens, Greece

**Keywords:** persistent organic pollutants, marine microorganisms, marine enzymes, biocatalysis, bioremediation

## Abstract

The aim of the present review is to highlight the potential use of marine biocatalysts (whole cells or enzymes) as an alternative bioprocess for the degradation of aromatic pollutants. Firstly, information about the characteristics of the still underexplored marine environment and the available scientific tools used to access novel marine-derived biocatalysts is provided. Marine-derived enzymes, such as dioxygenases and dehalogenases, and the involved catalytic mechanisms for the degradation of aromatic and halogenated compounds, are presented, with the purpose of underpinning their potential use in bioremediation. Emphasis is given on persistent organic pollutants (POPs) that are organic compounds with significant impact on health and environment due to their resistance in degradation. POPs bioaccumulate mainly in the fatty tissue of living organisms, therefore current efforts are mostly focused on the restriction of their use and production, since their removal is still unclear. A brief description of the guidelines and criteria that render a pollutant POP is given, as well as their potential biodegradation by marine microorganisms by surveying recent developments in this rather unexplored field.

## Marine Environment: Accessing a Hidden Biocatalytic Treasure

Bioprospecting of natural resources can be defined as the entire research and development processes that start from sample extraction funded by academia and research centers to full scale commercialization through, e.g., biotechnology companies (Leary et al., 2009). However, bioprospecting has been associated with both commercial (consumables, functional, future known, and future unknown values) and non-commercial values (benefit for future generations and cultural aesthetics; Armstrong et al., 2013). The term “natural resources” may refer to all kinds of organisms including animals, plants, and microorganisms, their genes, enzymes or secondary metabolites. The vastly expanding activities of biotechnology have created the need for new biocatalysts with diverse properties, being intended for different applications. Biocatalysts with varied properties of any kind (chemical, enantioselective, kinetic, stability) may find room or even create new paths in the field of biotechnology.

Marine environments, which cover over the two thirds of the earth’s surface, constitute a great pool of diversified natural resources, as they comprise more than 95% of the biodiversity of the total environment (Dalmaso et al., 2015) and they contain 178,000 species that represent 34 of the 36 living phyla (Dionisi et al., 2012; Dash et al., 2013). This broad biodiversity may be attributed to the broad spectrum of marine environments that are found on earth and can accommodate different types of life. Those marine habitats (coastal, pelagic, demersal) range between ice seas and hydrothermal vents, trenches and subsurface-formation habitats. These environments are dominated by different extreme conditions of light (sunlit surface to deep darkness), temperature (-35°C up to 350°C), and pressure (up to 110 MPa; Dionisi et al., 2012).

Microbes account for the 60% of the total biomass on earth and play a crucial part in sustainability (Dionisi et al., 2012). For 3.5 billion years, microbes have been accumulating mutations which led to high genetic and functional diversity (Sogin et al., 2006), rendering them a bottomless reservoir of genetic resources. Particularly, marine microorganisms, which represent more than 90% of the oceans’ biomass and produce over 50% of the earth’s oxygen (Dewapriya and Kim, 2014), are ubiquitous having been detected to all of the aforementioned extreme or mild marine habitats. Their diversity is considered to be a result of the high adaptation capability of microorganisms that react and acclimate promptly to their milieu (Dash et al., 2013). There has also been proposed that an individual bacterial species may consist of many different and definite sub-populations, existing side-by-side and displaying discrete characteristics (Seymour, 2014).

It has been calculated that approximately 3.6 × 10^29^ bacterial cells are present in the earth’s oceans, however, the number of species that they include is mostly important (Dewapriya and Kim, 2014). Up to now, it is thought that just a small fraction (1–10%) of prokaryotic species has already been characterized, mainly due to the fact that cultivation of marine microorganisms constitutes a great hindrance to this direction (Dalmaso et al., 2015). What has been epitomized in 1985 as “the great plate count anomaly” (Staley and Konopka, 1985), describes exactly this barrier. Approximately 1% of the microorganisms that are included in an environmental sample are cultivable in laboratory conditions. Despite the fact that most samples are dominated by some particular species (Seymour, 2014), in many cases these abundant taxa are the ones that cannot be cultured in the lab. On the other hand, rare species that cannot be detected easily by molecular techniques, are able to grow in laboratory conditions (Pedrós-Alió, 2006).

## Looking for Biocatalysts in the Blue Chest

In this paragraph, a brief description on the available scientific tools used to access novel marine-derived biocatalysts with potential biotechnological applications is carried out. For uncultivable microorganisms, the metagenomics approach is considered the best choice, while the isolation of the small fraction of cultivable microorganisms that are included in a marine sample is the key for accessing their diversity and enzymatic arsenal. The spotlight is set at marine microorganisms, such as bacteria and fungi and their enzymes, not just due to their biochemical diversity, but also because of their efficient biomass production and ease of genetic manipulation (Zhang and Kim, 2012).

### Metagenomics Approach

As it is already mentioned, only a tiny fraction (0.01–1%) of the microorganisms included in an environmental sample can be cultured by the conventional methods widely used. There is a well-established process for microorganisms that are considered uncultivable to access their genetic arsenal. Metagenomics, according to J. Handelsman, is “the genomic analysis of microorganisms by direct extraction and cloning of DNA from an assemblage of microorganisms” (Handelsman, 2004). In the last few years, many detailed review papers have been published on various aspects of the metagenomics approach for bioprospecting of biocatalysts and particularly for marine-derived ones (Kennedy et al., 2010, 2013; Kodzius and Gojobori, 2015; Vester et al., 2015). Concisely, as it is also depicted by Felczykowska et al. (2015), this process involves the extraction of the whole environmental DNA and in some cases its amplification and creation of a metagenomic library. Sample handling and DNA extraction method is a limiting step to the whole process, since it influences the final DNA amount and quality. Additionally, the choice of the cell lysis procedure has an impact on the sample adumbration, since different types of microorganisms (gram positive or negative bacteria, yeasts, and spores) exhibit distinctive sturdiness to the various disruption techniques (Kodzius and Gojobori, 2015). Subsequent cloning of the library in an appropriate host takes place, such as *Escherichia coli*, and then screening of the transformants for target activities. The screening could be either sequence-based or function-based.

The sequence-based screening is performed by either hybridization with labeled DNA probes or by PCR, using primers based on already known sequences. High throughput sequencing has been widely used, since it is affordable (Vester et al., 2015). DNA sequencing results are then screened by bioinformatics tools (Kennedy et al., 2010; Barone et al., 2014), which are also based on conserved sequences and a lengthy list of candidate genes is put together. This list is further screened and genes are prioritized regarding their theoretical properties and degree of novelty. The downside of sequence-based screening is that completely novel genes are most likely to be ignored by the process followed.

On the other hand, function-based screening of a metagenomic library is considered a lot harder as an approach involving tedious and slow procedures, but it has the opportunity to lead to the discovery of entirely novel biocatalysts. One hindrance of this procedure is the expression host, which in most cases is the *E. coli* system. The *E. coli* expression system faces some well-known difficulties at expressing foreign proteins, for example codon bias, misfolding of heterologous proteins, insolubility of the product, toxicity, and improper secretion. However, engineered *E. coli* strains are available expressing cofactors and chaperones or enhancing secretion of the recombinant protein (Vester et al., 2015). Alternatively, the use of other expression hosts, such as *Pseudomonas, Bacillus, Ralstonia, Agrobacterium, Burkholderia, Caulobacter, Streptomyces, Rhizobium* species, or the combination of hosts by using shuttle vectors may be employed (Kennedy et al., 2008, 2013; Troeschel et al., 2012). Additionally, since temperatures in 90% of all marine environment range from 5 to 10°C, the need for psychrophilic expression hosts is present (Pulicherla and Sambasiva Rao, 2013). An efficient expression and secretion system for psychrozymes has been developed using the Antarctic Gram-negative bacterium *Pseudoalteromonas haloplanktis*, which can produce extracellular recombinant proteins at temperatures as low as 4°C (Cusano et al., 2006). Furthermore, a commercially available *E. coli* strain (ArcticExpress by Agilent Technologies) harbors two cold-active chaperonin genes from the bacterium *Oleispira antarctica*, which help in protein folding at low induction temperatures. The final limiting step of function-based metagenomics is of course the screening method. The large number of metagenomic-library clones requires high throughput and sensitive screening techniques. In most cases, screening is performed by plate assays seeking for a positive clone that creates a clear halo or a change of color on the plate substrate depending on the enzymatic target activity (Popovic et al., 2015). The main drawback of this screening procedure is the limiting number of plate assays available. An alternative method for the detection of catabolic genes is the Substrate-Induced Gene Expression (SIGEX) screening, which relies on the fact that the genes of interest are induced by relative substances and their regulatory elements are usually located adjacent to them (Uchiyama and Watanabe, 2008). A similar approach is Product-Induced Gene Expression (PIGEX) screening, where the metagenomic library is cultivated along with reporter *E. coli* strains that detect the product of an enzymatic activity (Uchiyama and Miyazaki, 2010). Both SIGEX and PIGEX screenings are based on the detection of fluorescence in the presence of the widely used GFP protein, which is a marine-derived protein originating from the jellyfish *Aequorea victoria*.

Ferrer et al. (2016), who have provided a detailed analysis about the achievements of metagenomic bioprospecting, estimate that the majority of the enzymes discovered are esterases and lipases (68%) followed by glucosidases (16%) and oxidoreductases (14%). This result could be attributed to the well-established protocols for their functional screening. Furthermore, regarding lipolytic enzymes, 200 substrates have been used successfully for their screening, underpinning the need for the development of robust sensitive screening techniques for other enzymatic activities too. It is estimated that only 12% of all metagenomic surveys have concluded to the identification of actual enzymatic activities and of those cases functional screening has led to 30 times more discoveries than sequence screening (Ferrer et al., 2016). Out of the 12 marine-related libraries that have been constructed and screened with agar-plate assays, 545 positive hits have been identified for esterase/lipase, glycosyl hydrolase, protease, and dehalogenase activities (Popovic et al., 2015). In those cases, more than half of the discovered activities were putative esterases or lipases.

### Isolating Cultivable Microorganisms

Special attention must be paid toward culturing marine-derived microorganisms in order to access their diversity and enzymatic arsenal. Considering the high percentage of the uncultivable microorganisms, pure cultures of a microbial species must be obtained, aiming in understanding its role and importance in the ecosphere. Out of the enormous microbial diversity believed to be found in marine and terrestrial environments, only just a few thousand species have been described due to the inability to cultivate them in laboratory conditions.

There have been several cases of fungal and bacterial isolates from marine macroorganisms, such as invertebrates and algae, using conventional isolation methods. The general procedure involves wash (Baker et al., 2009; Henríquez et al., 2014) and/or sterilization of the macroorganism surface using sterile seawater and ethanol (Sponga et al., 1999) or mercury chloride in ethanol (Menezes et al., 2010; Kossuga et al., 2012). Inoculation of petri dishes can take place either by direct plating of sliced samples (Duarte et al., 2013) or by spreading them on the plates (Kossuga et al., 2012) or by diluted homogenized/triturated samples (Anand et al., 2006; Kossuga et al., 2012). There have been a lot of different media used for the isolation of fungi (Menezes et al., 2010) and bacteria (Skariyachan et al., 2014) prepared with seawater or artificial seawater that in case of fungal isolates was supplemented with an antibiotic [e.g., streptomycin (Duarte et al., 2013), benzylpenicillin (Henríquez et al., 2014), rifampicin (Menezes et al., 2010)] to avoid bacterial contamination.

Using the above mentioned techniques a great number of marine-derived strains has been isolated. Samples from various parts of the world, for example Antarctica, Ireland, Brazil, India, the Mediterranean, and Red Seas, where different conditions prevail have been studied. Typically, under standard laboratory conditions, 5–9 different species of fungi can be isolated from a macroorganism sample (e.g., from ascidians, cnidarians, sponges, algae, sea stars, urchins; Sponga et al., 1999; Da Silva et al., 2008; Duarte et al., 2013; Henríquez et al., 2014), while 4–7 strains per sample of marine sediments (Sponga et al., 1999; Duarte et al., 2013). However, in some cases reported, the number of isolates is increased dramatically. Menezes et al. (2010) and Kossuga et al. (2012) have managed to isolate 86 and 64 fungal strains, respectively, per sample of marine macroorganism including ascidians, sponges, and algae, by differentiating the isolation method. Additionally, Baker et al. (2009) isolated 80 fungal strains just from a single sponge strain of *Haliclona simulans*. The number of bacterial isolates per sample can range between 15 and 20 (Anand et al., 2006; Menezes et al., 2010). The sample plating method, the media and even the pigment agent used are important aspects of the isolation technique and have a substantial impact on the number and diversity of the resulting isolated strains. Different media can lead to the isolation of taxonomically different strains from the same sample. Furthermore, some species are common between different invertebrates, while others are found only in specific macroorganism species. Even by using these trivial isolation techniques, researchers have managed to identify species related to previously uncultured or unidentified ones or strains with very low similarity to known ones (Baker et al., 2009; Menezes et al., 2010; Henríquez et al., 2014).

Frequently used rich culturing media usually give the chance to fast-growing opportunistic microbes to grow. On the other hand, slow-growing microorganisms that are usually the most valuable due to the production of secondary metabolites beneficial in industry are rarely selected on these media. Therefore, the effort is driven toward the development of high throughput culturing methods, which will allow accessing the untapped microbial diversity of marine environment and broadening our knowledge and their potential use. There have been some recent review papers in reference to novel culturing techniques for the isolation and study of recalcitrant microorganisms (Stewart, 2012; Vester et al., 2015). Since we are not aware of the inability to culture most of the isolates, mimicking the natural environment as meticulously as possible might improve the possibility of growing such microbes, as well as transferring the natural environment itself inside the laboratory. Several methods have already been developed in order to handle the growth of pure cultures in a simulated natural environment. Diffusion (Kaeberlein et al., 2002) and hollow-fiber membrane (Aoi et al., 2009) chambers allow the growth of microbes in permeable compartments separate from the rest of the artificial environment, so as to remain uncontaminated while having access to nutrients and other growth factors. These techniques have been also developed in a high throughput fashion, aiming in the cultivation of a large number of separate microcolonies at the same time. A high throughput version of the diffusion chambers is the isolation chip (iChip) where hundreds of chambers can be operated simultaneously (Nichols et al., 2010). Moreover, a high throughput method was developed encapsulating microorganisms in gel microdroplets that are placed in a continuous fed-batch bed supplied with low nutrient flux, where metabolites can be exchanged between the microbes forming microcolonies (Zengler et al., 2002). Using the above mentioned techniques, the recovery rates of cultured microorganisms can increase dramatically, subsequently increasing the number of known microorganisms with novel biocatalytic potentials.

## Industrial Applications of Marine-Derived Biocatalysts

The global market for enzyme biocatalysts in industrial applications was about $4.8 billion in 2014 and it is expected to reach $7.1 billion by 2018 and $10 billion in 2020. Specifically, in the food/beverage industry and detergent markets, it is expected to reach a value of $1.7 billion and $1.8 billion by 2018, respectively (BCC Research, 2014). It is also believed that the investment in research and development around new psychrozymes will play an important role in the fast growth of the market.

Marine world is a particularly interesting place for the bioprospecting of novel biocatalysts. Marine-derived biocatalysts may possess appealing properties, acquired through millions years of evolution in unfriendly habitats, which render them valuable for biotechnological applications. These characteristics have been recently reviewed in detail by Dalmaso et al. (2015) and include tolerance in high salt concentrations (halophiles), high pressure (piezophiles), either high thermal (thermophiles) or cold adaptivity (psychrophiles), and combination of the above (polyextremophiles). Furthermore, some of the enzyme biocatalysts acquire novel chemical or stereochemical properties (Trincone, 2011), compared to their terrestrial counterparts, like substrate specificity and enantioselectivity, which can be exploited in organic synthesis and for the resolution of racemic mixtures with potential applications in the pharmaceutical industry.

It is reported that more than two thirds of the seawater’s volume has a constant temperature of 2°C (Russell et al., 1990). The microorganisms adapt to their cold milieu using several mechanisms, such as altering the composition of their cell membranes producing different fatty acids, mostly polyunsaturated and methyl-branched. Their cell membranes allow the preservation of the physical characteristics of the membrane, for example fluidity and permeability, retaining its functionality. Additionally, microbes produce cold shock, cold-acclimation, and antifreeze proteins in order to reassure the stability of RNA molecules (Pulicherla and Sambasiva Rao, 2013). Furthermore, cold-adapted microorganisms produce enzymes that can perform at such low temperatures and high viscosity. To achieve that, evolution has created enzymes with larger catalytic cavities and higher flexibility so as to overcome the low kinetic energy (Siddiqui and Cavicchioli, 2006).

Since the global trend is directed toward the discovery of novel psychrophilic biocatalysts, the potential applications of those will be discussed in more detail. The advantages of using cold-active biocatalysts in industrial processes are focused in the elimination of additional heating steps that leads to energy saving, prevention of contamination, thermal protection of sensitive reactants and products, especially in cases of volatile compounds and finally easy inactivation of the thermolabile catalysts by moderate heating. Marine-derived cold-active enzymes can be used in several industries; paper and textile (ligninases and proteases), biofuels production (cellulases, hemicellulases, and ligninases), cosmetics and pharmaceuticals (lipases and proteases). A promising market for cold-active enzymes like lipases, proteases, amylases, and cellulases, is obviously the detergent industry, where they are used as additives that boost the cleaning properties of the base detergent. Moreover, food and beverage industry has found room for the use of many cold-active enzymes considering the heat-sensitivity of the products, which distorts their organoleptic characteristics. Traditionally amylases may be used for the production of amyl syrups, cellulases for coffee processing, pectinases in fruit juices, β-galactosidases for milk processing and proteases for caviar production, fish descaling and squid skimming (Cavicchioli et al., 2002; Zhang and Kim, 2012; Pulicherla and Sambasiva Rao, 2013; Bonugli-Santos et al., 2015). Furthermore, except for enzymes, cold-adapted microorganisms can be employed for the production of antifreeze and ice-nucleation proteins that can be used as cryoprotectants or for synthetic snow generation. Marine microorganisms are valuable for the production of secondary metabolites that can be used as nutraceuticals or food additives, such as polyunsaturated fatty acids, pigments, and probiotics (Cavicchioli et al., 2002; Dewapriya and Kim, 2014).

A different application of marine-derived microorganisms could be the detoxification of wastewaters, decolorization of synthetic dyes and textile effluents and bioremediation of other recalcitrant pollutants such as persistent organic pollutants (POPs; Pulicherla and Sambasiva Rao, 2013; Bajaj and Singh, 2015; Bonugli-Santos et al., 2015; Rodriguez et al., 2015). Pollution by POPs often takes place in marine ecosystems and the exploitation of microorganisms might be a feasible alternative against typical mechanical methods.

## Marine Biocatalysts for Degrading PAHs and Halogenated Compounds

Naturally occurring aromatic compounds can be degraded by various microorganisms. The enzymes employed by these microorganisms to this end, can be used for the degradation of many man-made aromatic substances, included in pesticides, detergents, oils, solvents, paints or explosives (Dagley, 1975). The occurrence of chlorine substituents in many of these compounds increases their recalcitrance to enzymatic degradation, due to their reduced solubility and chemical reactivity (Reineke and Knackmuss, 1988). Consequently, chlorinated aromatic compounds are harder to degrade, as compared to their parent hydrocarbons.

In the following paragraphs, an overview of marine-derived biocatalysts involved in the degradation of aromatic and halogenated compounds is given. For the bioconversion of polycyclic aromatic hydrocarbons (PAHs), numerous marine microorganisms have been studied, while a limited number of enzymes, specifically dioxygenases, have been isolated and characterized. On the other hand, a number of marine-derived dehalogenases reported in literature, can be used in the biodegradation of recalcitrant pollutants.

### PAH Biodegradation

Polycyclic aromatic hydrocarbons, such as anthracene (C_14_H_10_), naphthalene (C_10_H_8_), phenanthrene (C_14_H_10_), and pyrene (C_16_H_10_), are a group of hydrophobic compounds composed of two or more fused aromatic rings that are formed by incomplete combustion of organic matter. Phenanthrene is a model PAH that contains three fused benzene rings in an angular arrangement, while naphthalene is the simplest PAH consisting of a fused pair of benzene rings. Naphthalene has long been used in enrichment cultures to isolate PAH-catabolizing bacteria.

Polycyclic aromatic hydrocarbons bioaccumulate through food chains and are known to exert toxic, mutagenic, teratogenic or carcinogenic properties on human health (Kanaly and Harayama, 2000). PAH degradation involves metabolic reactions catalyzed by a variety of enzymes, such as oxygenases, dehydrogenases, and ligninolytic enzymes (Haritash and Kaushik, 2009).

#### Metabolic Pathways for PAH Degradation

The first step of PAH degradation, which is the most crucial, involves the hydroxylation of the ring structure and is carried out by PAH dioxygenases, such as naphthalene or phenanthrene dioxygenase, which are three component enzymes, involving a ferredoxin, a ferredoxin reductase, and a terminal dioxygenase. Previous studies have shown that a single ferredoxin and a ferredoxin reductase can be shared by multiple dioxygenases (Mihyun, 2000).

In the second step of PAH degradation, the hydroxylated ring is cleaved oxidatively through either ortho cleavage (intradiol), leading to the generation of muconic acid or meta cleavage (extradiol), generating a hydroxymuconaldehydic acid derivative. The ring-cleavage reaction is catalyzed by catechol dioxygenases, which are non-heme iron-dependent enzymes, divided into intradiol and extradiol dioxygenases. The former, such as protocatechuate 3,4-dioxygenase (3,4-PCD) and catechol 1,2-dioxygenase (1,2-CTD), make use of a mononuclear non-heme Fe(III) cofactor for substrate binding and catalysis, while the latter utilize a mononuclear Fe(II) cofactor.

**FIGURE 1:**
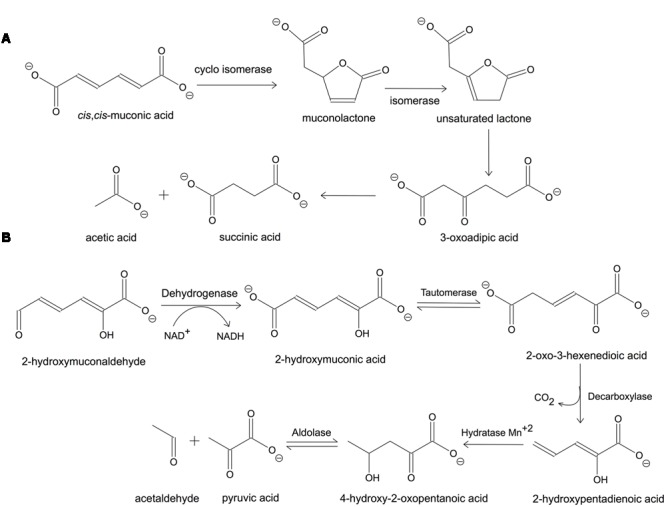
**Catechol degradation pathways by *Pseudomonas putida*. (A)** *Ortho*-cleavage and **(B)** *meta*-cleavage pathways. Adapted from Bugg and Winfield (1998).

The *ortho*-cleavage pathway for benzene in *Pseudomonas putida* is presented in **Figure 1A**. The *cis,cis*-muconic acid is cyclised enzymatically to give a five-membered lactone, muconolactone, which is then converted into the unsaturated lactone by an isomerase. The unsaturated lactone is subsequently hydrolyzed to 3-oxoadipic acid, which is finally cleaved to acetic and succinic acid (Bugg and Winfield, 1998). This pathway is used by *P. putida* for the degradation of chlorinated phenols as well. The meta-cleavage pathway can be completed in the following steps in *P. putida*: 2-hydroxymuconaldehyde is oxidized to give 2-hydroxymuconic acid, which is decarboxylated to 2-hydroxypentadienoic acid. The latter is further broken down by hydration to 4-hydroxy-2-oxopentanoic acid, followed by aldolase-catalyzed cleavage to acetaldehyde and pyruvic acid (**Figure 1B**) (Bugg and Winfield, 1998). Most catechol oxidations in nature occur via the extradiol pathway, however, these enzymes are less studied compared to intradiol dioxygenases due to their instability and dependence on ferrous ion [Fe(II)] and in some cases Mn(II).

Even though the majority of microbial aromatic cleavage pathways converge to catechol intermediates, some of them proceed via non-catecholic hydroxy-substituted aromatic carboxylic acids such as gentisate, salicylate, 1-hydroxy-2-naphthoate or aminohydroxybenzoate. These compounds are cleaved by extradiol dioxygenases, which belong to the cupin superfamily of proteins (Fetzner, 2012). The cupin superfamily is characterized by a conserved six-stranded β-barrel fold and its members consist of a single cupin domain (monocupins) or have duplicated domain structure (bicupins; Khuri et al., 2001). Dioxygenases belonging to this family involve gentisate 1,2-dioxygenase (GDO) as well as 1-hydroxy-2-naphthoate (HNDO) and salicylate 1,2-dioxygenases (SDO), cleaving their substrates between the adjacent carbon atoms carrying a carboxylate and a hydroxyl substituent (Fetzner, 2012).

#### PAH-Degrading Marine Microorganisms

In order to assess the effect of PAH accumulation in marine environments, there is need for appropriate bioindicators. Microbial populations and their reaction to environmental variations make a promising pollutant indicator. The microalga *Lingulodinium polyedrum* is a potential biomonitor for marine biota toxicity. Its enzymes, superoxide dismutase, and catalase, are induced in the presence of phenol, which stems from the decomposition of aromatic organic compounds, and can thus be used as biomarkers of increased antioxidant activity (Martins et al., 2015). Due to difficulties in cultivating natural bacteria, genetic fingerprinting can be used to generate the profile of microbial communities. Constructed 16S rRNA gene libraries showed that chronic PAH pollution in Black Sea coastal sediments resulted in an increase of α- and γ-Proteobacteria, whereas in non-polluted sites there was a prevalence of Actinobacteria (Todorova et al., 2014). It was suggested that α-Proteobacteria could serve as chronic oil pollution bioindicators.

Polycyclic aromatic hydrocarbon-degrading strains have been isolated from various marine environments, such as seawater, sediments, salt marshes, and estuaries. In their majority, they belong to the phyla Proteobacteria (α-, β- and γ-classes), Actinobacteria, Cyanobacteria, Bacteroidetes and Firmicutes (Louvado et al., 2015). Most studies observe decreased degradation efficiency with increasing PAH molecular weight (MW). This has been attributed to the decreased solubility and thus bioavailability of bigger compounds, but also to competitive inhibition, where more soluble PAHs repress the enzymes required to degrade high MW PAHs (Bacosa and Inoue, 2015). In addition, due to the toxicity of intermediate products released during PAH decomposition, their complete mineralization requires the synergistic effect of bacterial consortia (Cui et al., 2014; Gallego et al., 2014).

The γ-proteobacterial *Cycloclasticus* is considered as an obligate marine PAH degrader, with the potential to degrade PAH chlorinated derivatives as well (Yakimov et al., 2007). Phenanthrene- and naphthalene-degrading bacteria of the genus *Cycloclasticus* were isolated from the Gulf of Mexico by using a modified most-probable-number technique (Geiselbrecht et al., 1998). These bacteria can grow using PAHs, such as phenanthrene and naphthalene as sole carbon sources and they appear to be widespread in Pacific nearshore coastal environments. It was shown that in the presence of low MW PAHs that support cell growth, these *Cycloclasticus* strains can partially degrade higher MW PAHs. The conservation of the dioxygenase iron–sulfur protein (ISP) sequence indicates the importance of these enzymes in the *Cycloclasticus* lifestyle. *Pseudomonas monteilii* P26, *Pseudomonas stutzeri* N3, and *Pseudomonas xanthomarina* N12 isolated from contaminated Patagonian coasts were also shown to be capable of removing significant amounts of naphthalene and phenanthrene within 20 h, whereas pyrene was poorly degraded by all three strains (Isaac et al., 2015). Naphthalene dioxygenase activity was correlated with the expression of *nahAc* gene, encoding the iron–sulfur large (α) subunit of PAH dioxygenase. *Alteromonas* sp. strain SN2, detected in samples from contaminated sea-tidal flats from South Korea, was found able to degrade naphthalene, phenanthrene, anthracene, and pyrene. Naphthalene metabolism proceeded through the gentisate pathway. As anticipated, PAH degrading capacity decreased with increasing number of aromatic rings, and thus pyrene decomposition was very slow (Jin et al., 2012). The bacterium *Neptuniibacter* sp. strain CAR-SF, isolated from seawater, was capable of using carbazole, a common environmental pollutant usually originating from coal tar, as sole carbon and nitrogen source. Carbazole degradation occurred through two pathways, the upper and lower, each one operated by a distinct cluster of genes, similar to their terrestrial counterparts (Nagashima et al., 2010).

It has been shown that marine phytoplankton can adsorb PAH molecules, providing a favorable niche for PAH-degrading bacteria. The γ-proteobacterial strain TG408 was isolated from marine phytoplankton and displayed a narrow nutritional spectrum. It was shown to preferably utilize aliphatic and aromatic hydrocarbon compounds, and small organic acids. Strain TG408 is strictly aerobic, and can grow at temperatures as low as 10°C. It is catalase and oxidase positive and degrades naphthalene via the meta-cleavage pathway (Gutierrez et al., 2013).

Due to their hydrophobic and lipophilic nature, PAHs adhere to soil particles in a seawater environment. During, e.g., oil spills, small chain alkanes (<C14) and low MW PHAs are removed by physical weathering, while long chain and branched alkanes are removed by biodegradation. The rest, including high MW PAHs, will tend to accumulate to the sediment (Louvado et al., 2015). Deep sea sediments that refer to the sea floor at depths below 1000 m, are characterized by high pressure (10–50 Mpa), low temperatures (2–3°C) and low concentration of labile organic carbon. Increased salinity favors PAH adherence to soil particles and thus reduces their bioavailability. Low temperature and darkness result in lower metabolic activity of PAH-degrading microorganisms (Marini and Frapiccini, 2013), while high pressures decrease bacterial growth and PAH degradation efficiency (Schedler et al., 2014). PAH-degrading microorganisms that have been isolated from deep sea environments involve α- and γ-Proteobacteria, and to a lesser extent β-Proteobacteria, Actinobacteria, and Flavobacteria (Louvado et al., 2015). PAH-degrading bacteria have been isolated from tsunami sediments as well. Tsunami sediments display unique features that could be exploited to identify novel microbial populations involved in PAH degradation. The bacterial consortia had the ability to degrade fluorene and phenanthrene, while pyrene was the least degraded compound. They were dominated by known PAH degraders such as *Sphingomonas, Pseudomonas*, and *Sphingobium*, as well as unknown genera, such as *Dokdonella* and *Luteimonas* (Bacosa and Inoue, 2015).

#### Marine Enzymes in PAH Degradation

A limited number of marine-derived dioxygenases have been isolated and characterized (**Table 1**). The phenanthrene-degrading *Nocardioides* sp. strain KP7 was isolated from a Kuwait beach based on its detoxification capacity a couple of years after the occurrence of an oil spill accident (Iwabuchi and Harayama, 1998). HNDO, purified from strain KP7, is an enzyme that oxidizes 1-hydroxy-2-naphthoate, which is an intermediate product of phenanthrene bacterial degradation pathway. This enzyme is unique, since it can cleave a singly hydroxylated aromatic ring. In specific, it cleaves the aromatic ring between the carboxylated and hydroxylated carbon of the substrate. It is a homohexamer in solution, with a *K*_m_ of 10 μM and *k*_cat_ of 114 s^-1^ at 25°C when 1-hydroxy-2-naphthoate is used as a substrate, and displays a pH_opt_ of 7.5 and T_opt_ of 40°C. It is inactivated by 0.1 mM *o*-phenanthroline that is a specific chelator of the enzyme cofactor Fe(II).

**Table 1:** Examples and properties of marine-derived dioxygenases and dehalogenases.

Enzyme	Activity	Source	MW (kDa)^1^	T_opt_	Reference
Dioxygenase	1-hydroxy-2-naphthoate dioxygenase	*Nocardioides* sp. strain KP7	45	40	Iwabuchi and Harayama, 1998
	PhdABCD dioxygenase	*Nocardioides* sp. strain KP7	n.d.	n.d.	Saito et al., 2000
Dehalogenase	DrbA HLD	*Rhodopirellula baltica* SH1	n.d.	50	Jesenská et al., 2009
	DppA HLD	*Plesiocystis pacifica* SIR-1	35	33–37	Hesseler et al., 2011
	DmmA HLD	*Moorea producta*	n.d.	n.d.	Gehret et al., 2012
	HanR HLD	*Rhodobacteraceae*	n.d.	n.d.	Novak et al., 2014
	DadB HLD	*Alcanivorax dieselolei* B-5	34.2	50	Li and Shao, 2014
	DpcA	*Psychrobacter cryohalolentis* K5	n.d.	25	Drienovska et al., 2012
	Deh99 HAD	*Paracoccus* sp. DEH99	25	40	Zhang et al., 2014
	HAD I	*Pseudomonas stutzeri* DEH130	109.9	n.d.	Zhang et al., 2013
	HAD II	*P. stutzeri* DEH130	26	40	Zhang et al., 2013
	DehRhb HAD	*Rhodobacteraceae*	25	55	Novak et al., 2013a
	PinHAD	*Psychromonas ingrahamii*	25	45	Novak et al., 2013b

*^1^Determined by SDS-polyacrylamide gel electrophoresis. Abbreviations: n.d., not determined; HLD, haloalkane dehalogenase; HAD, 2-haloacid dehalogenase.*

The gene from *Nocardioides* sp. strain KP7 that encodes phenanthrene dioxygenase (*phd*) has also been characterized (Saito et al., 2000). This enzyme catalyzes the first step in phenanthrene catabolism, being responsible for the conversion of phenanthrene to *o*-phthalate. The ring hydroxylating dioxygenase is composed of four components, PhdA, -B, -C, and -D, which were cloned and expressed in *E. coli.* PhDA and B show homology to the α- and β subunit of known ring-hydroxylating dioxygenases. PhdC exhibits significant homology to the [3Fe-4S] and [4Fe-4S] ferredoxins, and PhdD shows similarity to proteins of the reductase family. All four components are required for the full activity of PhdABCD dioxygenase. In the case of heterologous expression in *E. coli*, the ferredoxin component PhdC and the reductase component PhdD were not necessary for full activity, probably because of the fact that they were replaced by other *E. coli* electron transport proteins. This result has also been observed for other ring-hydroxylating dioxygenases, reflecting a tolerance between the oxygenase components and electron transfer systems. The *phdabcd* gene cluster from strain KP7 was subsequently introduced into *Streptomyces lividans*, a soil prokaryote that does not participate in PAH biodegradation (Chun et al., 2001). Recombinant *S. lividans* cells expressed the genes effectively and hydroxylated phenanthrene. They also transformed 1-methoxynaphthalene to its diol, 8-methoxy-1,2-dihydro-1,2-naphthalenediol, which is then non-enzymatically converted to 8-methoxy-2-naphthol. The recombinant strain could be used for bioremediation of soil environment polluted with PAHs.

### Degradation of Halogenated Compounds

Dehalogenation is a critical step in the biodegradation of halogenated compounds. The carbon–halogen bond can be cleaved either by enzymatic dehalogenation or by spontaneous dehalogenation of unstable intermediates. When it comes to enzymatic degradation, there are seven mechanisms: (1) hydrolytic dehalogenation, catalyzed by halidohydrolases; (2) reductive dehalogenation, which is catalyzed by reductive dehalogenases; (3) oxygenolytic dehalogenation, catalyzed by mono- or dioxygenases; (4) thiolytic dehalogenation, catalyzed by glutathione *S*-transferase (GST) enzymes; (5) intramolecular substitution, catalyzed by halohydrin–hydrogen halide lyases also called halohydrin epoxidases; (6) dehydrohalogenation catalyzed by dehydrohalogenases; and (7) hydration (Fetzner and Lingens, 1994; Bhatt et al., 2007). In the following paragraphs, after a brief overview of the enzymatic activities involved in the first two mechanisms, we will focus on dehalogenating biocatalysts originating from marine environments.

#### Hydrolytic and Reductive Dehalogenation

Hydrolytic dehalogenation proceeds via the replacement of the halogen with a hydroxyl group derived from water. There is a broad range of hydrolytic dehalogenases involved in the mineralization pathways of halogenated compounds, that have fundamentally different catalytic mechanisms (Janssen et al., 2005).

Haloalkane dehalogenases (HLDs, E.C. 3.8.1.5) catalyze the hydrolytic cleavage of carbon–halogen bonds for the dehalogenation of haloalkanes. They are members of the α/β superfamily and their active site is situated between a conserved core domain and a more variable lid domain. The most functionally important amino acids involve a catalytic triad consisting of the key nucleophile (Asp), the general base (His) and a catalytic acid, and two hydrogen bond-donating residues that stabilize the halide leaving group (Janssen, 2004). The dehalogenase reaction is completed in two steps (Verschueren et al., 1993).

2-Haloacid dehalogenases (HADs, E.C. 3.8.1.2) catalyze the dehalogenation of 2-alkanoic acids to produce 2-hydroxyalkanoic acids. L-2-haloacid dehalogenases (L-HADs) act on L-2-haloalkanoic acids to produce the corresponding D-2-hydroxyalkanoic acids; D-2-haloacid dehalogenases (D-HADs) act on D-2-haloalkanoic acids to produce the corresponding L-2-hydroxyalkanoic acids. In addition, DL-2-haloacid dehalogenases/configuration inversion (DL-HADis) dehalogenate L- and D-2-haloalkanoic acids to the corresponding D- and L-2-hydroxyalkanoic acids, and DL-2-haloacid dehalogenases/configuration retention (DL-HADrs) dehalogenate L- and D-2-haloalkanoic acids to the corresponding L- and D-2-hydroxyalkanoic acids (Soda et al., 1996).

Up to now, two hydrolytic dehalogenases targeting halogenated aromatics have been reported, acting on 4-chlorobenzoate (4-CBA) and 2,4,5,6-tetrachloroisophtalonitrile (chlorothalonil, TPN), respectively. 4-chlorobenzoyl-coenzyme A (CoA) dehalogenase (EC 3.8.1.7), the first reported member of this family, participates in the hydrolytic substitution of the chlorine atom of 4-CBA by a hydroxyl group and the generation of 4-hydroxybenzoate (4-HBA). This reaction requires two additional enzymatic activities, a ligase and a thioesterase, as well as the cofactors ATP and CoA (Scholten et al., 1991; Schmitz et al., 1992). The second reported enzyme is chlorothalonil hydrolytic dehalogenase (Chd) from *Pseudomonas* sp. CTN-3. Chd catalyzes the removal of the 4-chlorine atom from this highly toxic compound, generating 4-hydroxy-2,5,6-trichloroisophthalonitrile (4-TPN-OH). Unlike 4-chlorobenzoate dehalogenase, Chd does not require any additional cofactor and differs in terms of both sequence and function from other known dehalogenases (Wang et al., 2010).

Reductive dehalogenation proceeds via the replacement of a halide ion by a hydrogen atom and requires the transfer of two electrons. The role of electron donor can be fulfilled by a reduced organic substrate or H_2_. If the donor has sufficient reducing capacity, it provides the required electrons and single proton and the dehalogenation reaction is completed in one step. Alternatively, the donor provides the two electrons and the proton is offered by another source such as water, and the reaction is completed in two steps (Habash et al., 2004).

Even though it was initially thought that reductive dehalogenation is mainly carried out by anaerobic microorganisms, it can also occur under aerobic conditions. The soil bacterium *Sphingomonas chlorophenolica* can degrade pentachlorophenol (PCP), a xenobiotic pesticide, using a recently evolved pathway that involves a flavin monooxygenase, a reductive dehalogenase and a ring-cleaving dioxygenase. The first enzyme hydroxylates PCP to tetrachlorohydroquinone, which is then converted to trichloroquinone by a reductive dehalogenation reaction catalyzed by tetrachlorohydroquinone dehalogenase (TCD), a member of the zeta class of GST superfamily (Copley, 2000). Another case of reductive dehalogenation under aerobic conditions is the degradation of the brominated aromatic herbicide bromoxynil by *Comamonas* sp. 7D-2. The enzymatic players implicated in this pathway include a nitrilase, a reductive dehalogenase (BhbA), a monooxygenase, and a protocatechuate dioxygenase. BhbA is composed of a respiration-linked dehalogenase domain and a NAD(P)H-dependent oxidoreductase domain. It exhibits some common characteristics with anaerobic respiratory reductive dehalogenases, such as the association with the cell membrane and the presence of Fe-S cluster binding motifs. Interestingly, almost all of its sequence homologs are found in marine aerobic proteobacteria (Chen et al., 2013).

#### Dehalogenating Biocatalysts Isolated from Marine Sources

A number of dehalogenases that have been isolated and characterized from marine microorganisms are presented in **Table 1**, and their properties are briefly described in the following paragraphs:

##### Family I HLDs

HLD DppA from *Plesiocystis pacifica* SIR-1, a marine myxobacterium isolated from Japanese coast, is a 35 kDa enzyme with activity against short and medium chain α-bromoalkanes and short α,ω-bromoalkanes, but unable to degrade chloroalkanes. Structurally, it is similar to other known HLDs, consisting of two domains, a core domain with the canonical α/β hydrolase fold and a helical cap domain. It retains 75% of its maximum activity between 25 and 45°C and most of its activity between pH 7 and 9.5 (Hesseler et al., 2011).

DpcA is a family I HLD from *Psychrobacter cryohalentis* K5, a psychrophilic bacterium isolated from saline-water lenses derived from a 40,000-year-old Siberian permafrost. It has a melting temperature of 34.7°C, which is indicative of low thermal stability in comparison to related enzymes, and displays a narrow specificity profile, with preference toward longer substrates (number of carbons ≥ 3) and specifically 1-bromobutane, 1-bromohexane and 1,3-dibromopropane. Its overall dehalogenase activity is moderate when compared to other HLDs. Using 1-bromobutane as substrate, the highest activity of the enzyme is observed at 25°C and pH 8.7.

##### Family II HLDs

DmmA is a family II HLD identified and characterized from the metagenomic study of DNA of a *Moorea producta* field isolate (Gehret et al., 2012). *M. producta* is a marine cyanobacterium, however. DmmA should originate from a symbiont or associated bacterium. Its crystal structure revealed a wider entrance tunnel and substrate cleft, allowing the accommodation of bulkier substrates, as compared to other HLDs. Activity measurements over a range of substrates, showed that DmmA displayed higher preference for the bulky bromohexane over corresponding linear molecules, suggesting that the natural DmmA substrate could be a halogenated ring system. This property renders DmmA a valuable biotechnological tool (Gehret et al., 2012). Another studied family II HLD is HanR from a *Rhodobacteraceae* species isolated from the surface of a tube worm. HanR is active against both chloro- and bromo-alkanes, with preference for longer chains. Its substrate preference can be attributed to a more expanded space in the substrate binding channel, as evidenced by its native and complex crystal structures. In addition, a positively charged arginine residue at the bottom of the active site, which is not observed in other HDLs, facilitates the binding of distal halogen groups (Novak et al., 2014).

DadB from *Alcanivorax dieselolei* B-5, an important marine oil-degrading bacterium, is active against a variety of halogenated compounds, with preference against short chains, brominated alkanes, and chlorinated alkenes (Li and Shao, 2014). It can degrade the persistent environmental pollutants 1,2-dichloroethane, 1,2-dichloropropane, and 1,2,3-trichloropropane, underpinning the potential of *Alcanivorax* bacteria as cleaners of contaminated seawater. In general, DadB displays broader substrate specificity when compared to known HLDs, which could be explained by a large active site cavity, with the potential to accommodate a broad range of substrates.

##### HADs

The first report on the isolation and characterization of a HAD from a marine bacterium was that of two HADs from *Pseudomonas stutzeri* DEH130 (Zhang et al., 2013). Dehalogenase I is a 109.9 kDa enzyme that preferentially degrades D-2-chloropropionate with a pH optimum of 7.5. Dehalogenase II is a dimeric enzyme with a MW of the monomer of 26 kDa, and preferentially degrades L-2-chloropropionate with a *K*_m_ of 0.3 mM, and a pH and T optimum of 10.0 and 40°C, respectively. Its activity is strongly inhibited by Cu^2+^, Zn^2+^, and Co^2+^, but remains unaffected by DTT and EDTA.

Deh99A is a HAD purified from the marine bacterium *Paracoccus* sp. DEH99, isolated from the marine sponge *Hymeniacidon perlevis*. It is a 25 kDa enzyme that exists as a dimer in solution. It can stereospecifically dehalogenate L-2-chloropropionate to produce D-lactate, with a *K*_m_ of 0.21 mM, and a pH and T optimum of 10.0 and 40°C, respectively. Deh99 is strongly inhibited by Cu^2+^ and Zn^2+^ (Zhang et al., 2014).

Eleven bacterial strains isolated from the marine sponge *Hymeniacidon perlevis* degraded 2-chloropropionic acid (2-CPA; Huang et al., 2011). Based on the 16S rRNA gene sequence analysis, they were clustered into the *Rhodobacteraceae* family of α-Proteobacteria and the *Pseudomonadaceae* family of γ-Proteobacteria. In a subsequent report, one of the L-HADs from the *Rhodobacteraceae* family, named DehRhb, was cloned and overexpressed in *E. coli* (Novak et al., 2013a). Similar to previously studied L-HADs, it is a dimer in solution with a monomer MW of 25 kDa. It is active on a broad range of L-2-haloalkanoic acids, of varying chain lengths and halogen substitution, with highest activity against monobromoacetic acid. Using the latter as a substrate, T optimum was determined to be 55°C, while *V*_max_ and *K*_m_ were 1.75 μM min^-1^ mg^-1^ of protein and 6.72 mM, respectively. It displayed moderate thermal stability, however, it was highly tolerant to solvents. The crystal structures of DehRhb revealed an active site with significant differences from previously studied L-HADs, in terms of water activation, rendering it a novel member of the L-HAD family (Novak et al., 2013a).

There are limited reports on enzymes originating from microorganisms living in the sea ice and its interface with water. *Psychromonas ingrahamii*, isolated from the sea ice interface, is a marine bacterium with lowest growth temperature of -12°C (Breezee et al., 2004). The recombinant expression and characterization of the L-HAD PinHAD from *P. ingrahamii* showed that in spite of originating from a psychrophilic organism, the enzyme has mesophilic properties with an optimal temperature for activity of 45°C. It displayed the highest activity against bromoacetic acid, with a *V*_max_ and *K*_m_ of 0.6 μM min^-1^ mg^-1^ of protein and 1.36 mM, respectively (Novak et al., 2013b). It was generally more active against short chain (<C3) haloacids, without discriminating between chlorine and bromine in the α-carbon position, and exhibited a relative stability in organic solvents, in comparison to other characterized L-HAD enzymes.

##### Reductive dehalogenases

Reductive dehalogenation is commonly used by bacteria under the anoxic conditions that occur some centimeters below the surface of marine sediments. These environments constitute the ultimate sink for persistent man-made pollutants, such as polychlorinated biphenyls (PCBs), polychlorinated dibenzo-*p*-dioxins (PCDDs), polybrominated diphenylethers (PBDEs), and 1,1,1-trichloro-2,2-bis(*p*-chlorophenyl)ethane (DDT). In the case of compounds such as PCBs, the main factors limiting dechlorination efficiency involve the increased sulfate concentration competing for electron donors or directly inhibiting dehalogenation, the low availability of contaminants as substrates for bacterial growth and the small amount or inefficiency of indigenous dehalorespirers (Zanaroli et al., 2015). One of the measures taken to overcome some of these obstacles and increase dechlorination efficiency, is to enrich sediments with alternate halogenated compounds (Ahn et al., 2007). Another approach for *in situ* enhancement of reductive dehalogenation of organohalides in marine and estuarine sediments is bioaugmentation. This approach was successfully implemented in estuary sediment mesocosms supplemented with *Dehalobium chlorocoercia* DF-1, resulting in an increase in the degree of PCB dechlorination (Payne et al., 2011). Efforts are currently oriented toward the development of a PCB degrader that can tolerate higher salinity and higher sulfate concentrations, in order to implement the bioaugmentation approach in marine sediments as well (Zanaroli et al., 2015).

## What Makes a Pollutant Considered as POP?

**FIGURE 2:**
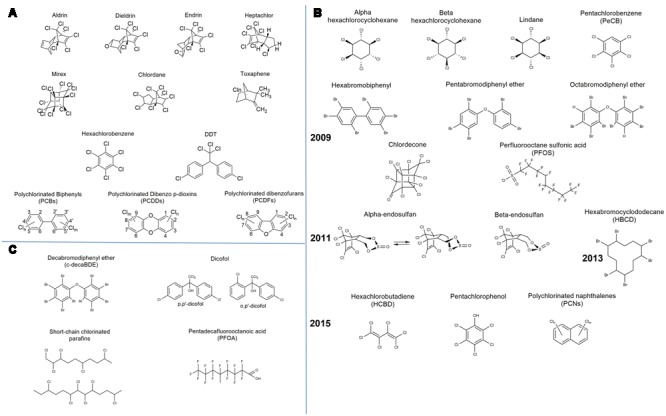
**Chemical formulae of the listed persistent organic pollutants (POPs) by the Stockholm Convention: (A)** “The Dirty Dozen,” **(B)** the new POPs and the year they were listed, and **(C)** new proposals to be reviewed.

Persistent organic pollutants have been well documented since the well-known Stockholm Convention of the United Nations Environment Programme (UNEP) that took place in 2001. Initially, the Stockholm Convention considered as POPs the notorious “Dirty Dozen” (**Figure 2A**) that had already been identified as harmful for the human health and the ecosystem (Ritter et al., 1995). Nonetheless, POPs were acknowledged for a long time before this particular convention. A concise history about the discovery and use of POPs along with the legislation and actions around them is given by Megson and O’Sullivan (2013). The works of the Stockholm Convention, however, have further plainly defined the criteria and proceedings for upgrading a substance to the POP category and listing it under Annexes A (elimination), B (restriction), and/or C (unintentional production) (Final Act of the Conference of Plenipotentiaries on the Stockholm Convention on Persistent Organic Pollutants, 2001). The main prerequisites for a substance to be considered into one of the above annexes are to be persistent, bioaccumulative, to have the ability of long-range transport and adverse effects on human health (Final Act of the Conference of Plenipotentiaries on the Stockholm Convention on Persistent Organic Pollutants, 2001).

For each of these criteria, certain constraints must be met for a chemical in order to be considered as a candidate POP. Half-lives of more than 2 days in air and 2–6 months in water, soil or sediments are considered sufficient for a candidate POP (Wahlström, 2003). Bioaccumulation is the ability of POPs to be absorbed by living organisms and due to their hydrophobic nature they tend to accumulate in adipose tissues. Consequently, further distribution of POPs can take place through food webs (Van Ael et al., 2013), even though some researchers have suggested that the equilibrium concentrations in living organisms are independent of predation links of different trophic levels but dependent of the lipid content of each organism (Xia et al., 2015). The ability of POPs to persist degradation and their long half-lives grant them the potential for long-range transport, at formerly pristine areas with no original POPs sources (Zhao et al., 2012). However, the transport of these chemicals does not take place only through water, but also through the atmosphere, contaminating remote sites and mountain zones (Ruiz-Fernández et al., 2014; Catalan, 2015; Lammel, 2015; Zhao et al., 2015), making POPs a world-wide issue. Humans’ intake of POPs is mainly occurring through their diet (Chen et al., 2012) and particularly through the consumption of animal-derived food (Gasull et al., 2011). Seafood (Perelló et al., 2015) from contaminated sources and especially fish consumption (Deribe et al., 2014; Lee S.-H. et al., 2014; Squadrone et al., 2014; Sun et al., 2014) take part in the human intake of POPs. Other potential human sources of POPs could be meat (Hernández et al., 2015) and vegetables (Khan and Cao, 2011), while POPs can be transferred through maternal milk to the infant (Croes et al., 2012; Chen et al., 2015) exposing it to detectable amounts of POPs in the very early stages of life. Infant exposure to such chemicals, either prenatal or postnatal, has been associated with several health issues ranging from growth (Iszatt et al., 2015) and obesity (Tang-Péronard et al., 2014; Vafeiadi et al., 2015) disorders to high blood pressure (Vafeiadi et al., 2015), and effects on the development of nervous (Berghuis et al., 2015), immune, and respiratory (Gascon et al., 2013) systems. In addition to infant population, POPs cause several adverse health effects in adults. Exposure to POPs has been mainly associated with carcinogenesis (Boada et al., 2012; Hernández et al., 2015; Lim et al., 2015; Mitro et al., 2016) and metabolic diseases (Ruzzin, 2012; Lee D.-H. et al., 2014) such as obesity (Dirinck et al., 2010; Lee et al., 2012; Reaves et al., 2015) and type two diabetes (Jaacks and Staimez, 2015; Ngwa et al., 2015), while numerous links with negative health consequences have also been reported (Crinnion, 2011; Lind et al., 2012; Kvist et al., 2014; Vested et al., 2014; Arrebola et al., 2015; Grindler et al., 2015).

Chemicals that possess the aforementioned characteristics, such as persistence, bioaccumulation, long-range environmental transport, and toxicity, need to firstly be proposed to the Persistent Organic Pollutants Review Committee (POPRC) in order to be listed as new POPs. The POPRC then drafts a risk profile and evaluates the possible global effects of a target chemical. Subsequently, a proposal is made to the Conference of the Parties (COP), which is the governing body of the Stockholm Convention that evaluates the proposal and lists the particular chemical to one of the Annexes A, B, and/or C. The POPRC was established in 2005 and since then holds meetings annually in Geneva or Rome. Additional to the initial 12 POPs, the COP has approved POPRC recommendations for 11 new chemicals, nine in 2009, one in 2011, and one in 2013 (**Figure 2B**). New proposals that have been submitted to the POPRC and are currently under review are also presented in **Figure 2C**. Further information about the actions, initiatives, and decisions taken by the Stockholm Convention can be found in its official website^1^.

## POP Biodegradation By Marine Biocatalysts

Marine sediments constitute a reservoir for POPs such as DDT, hexachlorocyclohexane (HCH), PCB, and PCDD. Their degradation is controlled by both abiotic and biotic processes. A metagenomic analysis identified a consortium of microorganisms carrying many biodegradation genes, with the potential to be applied for the mineralization of POP compounds. Most of the detected genes belonged to the Proteobacteria, followed by the Actinobacteria. More specifically *Plesiocystis*, which belongs to the Proteobacteria phylum was the most abundant genus in all annotated genera, followed by *Anaerolinea* (Chloroflexi phylum), *Jannaschia* (Proteobacteria phylum), *Mycobacterium* (Actinobacteria phylum), and *Pseudovibrio* (Proteobacteria phylum; Fang et al., 2014).

**FIGURE 3:**
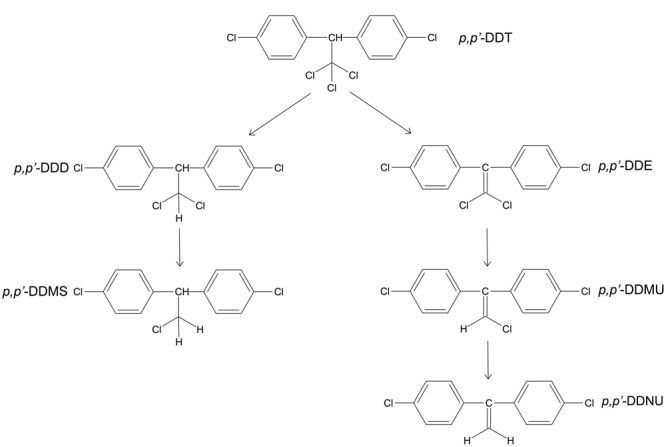
**Proposed degradation pathways of 1,1,1-trichloro-2,2-bis-(*p*-chlorophenyl)ethane (*p,p’*-DDT).** Abbreviations: *p,p’*-DDD, 1,1-dichloro-2,2-bis(*p*-chlorophenyl)ethane; *p,p’*-DDMS, 1-chloro-2,2-bis(*p*-chlorophenyl)ethane; *p,p’*-DDE, 1,1-dichloro-2,2-bis(*p*-chlorophenyl)ethylene); *p,p’*-DDMU, 1-chloro-2,2-bis(*p*-chlorophenyl)ethylene; *p,p’*-DDNU, 2,2-bis(*p*-chlorophenyl)ethylene. Adapted from Yu et al. (2011).

The microbial reductive dechlorination of DDT proceeds via the dechlorination of the aliphatic chloroethyl group of the molecule. It has been shown to involve reductive dechlorination, hydrogenation, dioxygenation, hydroxylation, decarboxylation, hydrolysis, and meta-ring cleavage reactions. The enzymes implicated in these reactions involve dehydrochlorinase, dioxygenase, reductase, decarboxylase, and hydrolase (Fang et al., 2014). The main steps in potential DDT degradation pathways were described based on analyses of marine sediment samples from of the Chinese coastal area (**Figure 3**) (Yu et al., 2011).

The bacteria that mediate the dechlorination of PBDEs and DDT in microbial communities of marine or estuary origin have not been identified yet. In the case of PCB, dehalorespiring microorganisms cluster with or are very close to the non-*Dehalococcoides* lineage of the class of Dehalococcoidia, which is represented by *D. chlorocoercia* DF-1 (Zanaroli et al., 2015). PCDD dechlorination in estuarine sediments has been associated with *Chloroflexi*-like microorganisms related to *Dehalococcoides* (Ahn et al., 2007). HCH degradation pathway includes dechlorination, hydroxylation, dehydrogenation, and phenyl ring cleavage reactions with dehydrochlorinase, dehalogenase, dehydrogenase, dechlorinase, and 1,2-dioxygenase from several bacteria such as *Mycobacterium, Sphingobium, Bradyrhizobium, Burkholderia*, and *Ralstonia* (Fang et al., 2014).

One of the most characterized POP degraders is *Sphingomonas wittichii* RW1 [DSM6014], which is capable of mineralizing polychlorinated dibenzo-*p*-dioxins/furans (PCDD/Fs). Originally isolated from a river in Germany, this strain first hydroxylates the chlorinated dibenzo-*p*-dioxins/furans (DD/Fs) and subsequently cleaves the resulting *ortho*-hydroxylated rings (Keim et al., 1999). Both reactions are catalyzed by dioxygenase enzymes. Even though this strain mineralizes fast DD/F, i.e., the carbon backbone of PCDD/Fs, chlorination results in decreased conversion rates. The effect of decreasing degradation efficiency with increasing halogen substitution has been also observed for other POP degraders. This may be due to steric inhibition or increased hydrophobicity of highly substituted POPs. In addition, the catabolism of POP compounds can lead to toxic end-products, like in case of PBDEs, which are converted by *Sphingomonas* sp. PH-07 to bromophenols and bromocatechols (Jeon et al., 2016).

In order to increase POP degradation efficiency, bacteria form partnerships with fungi and plants. The enhancement resulting from such beneficial interactions is accomplished through complementary catabolic reactions but is also due to morphological factors, such as the fungal hyphae that facilitate the adsorption of hydrophobic compounds (Boonchan et al., 2000; Arriaga and Revah, 2005). For example, the marine plant *Myriophyllum aquaticum* releases oxidation products of 2,4,6-trinitrotoluene (TNT) that have fewer nitro groups and are thus more susceptible to bacterial degradation (Ramos et al., 2005). The cooperation among bacterial populations can also result in improved POP degradation, due to variability in carbon source preference among the different strains (Vinas et al., 2005).

Aerobic bacterial catabolism can be modulated by abiotic agents as well. Zero-valent irons (ZVIs) and bimetallic ZVIs such as Pd/ZVI can assist POP degradation by removing halogen ions that confer hydrophobicity and steric inhibition to bacterial enzymes (He et al., 2009). Dehalogenation by ZVI nanoparticles takes place under anoxic conditions, followed by aerobic bacterial catabolism of the dehalogenated compound. The efficiency of this process can be optimized after considering two issues: (a) the toxicity of the nanoparticles to the bacteria and (b) the presence of oxygen which can generate reactive oxygen species (ROS) by ZVIs, resulting in the decomposition of bacterial catabolic proteins (Jeon et al., 2016). Another way to optimize bacterial POP degradation is by implementing advanced oxidation processes (AOPs) such as photocatalysis, ozonation, and Fenton chemistry. AOPs generate hydroxyl radicals that modify POPs in a way that their further bacterial catabolism is facilitated. In specific, AOPs generate hydroxylated or ester and carboxyl group-containing compounds that are less hydrophobic than their precursors, while could also oxidatively dehalogenate POPs (Jeon et al., 2013). Finally, one of the main obstacles to efficient soil bioremediation is the limited contact between microorganisms and pollutants, resulting from the heterogeneity of the solid matrix, and the immobilization of microorganisms. It has been shown that weak electric field application in soil can enhance bacterial mineralization of aromatic and/or halogenated compounds, by homogenizing microorganisms and pollutants in soil (Harms and Wick, 2006).

## Conclusion

Ocean is the place on earth where life first started, therefore it has the biggest potential for the discovery of novel biocatalysts with industrial interest. The increasing pollution by toxic compounds that resist biodegradation, has given rise to the exploration of nature’s wealth for the discovery of innovative bioremediation processes. Identification of the aromatic pollutant degrading potential from marine-derived microorganisms is a growing field, however, to date, only a limited number of biocatalysts have been discovered. In this review paper, a comprehensive presentation of the enzymatic toolbox and the corresponding mechanisms involved in the degradation of aromatic and halogenated compounds is carried out. In addition, information on POPs and description of the available scientific tools to access novel marine-derived biocatalysts capable of degrading these pollutants was given. Certainly, there are many unexplored microorganisms and enzymes originating from marine environments that could be used in bioremediation processes. In spite of the aforementioned difficulty in isolating a broad spectrum of marine microbes and their enzymes, some recent achievements have demonstrated their considerable potential in the detoxification of polluted environments, highlighting the need for further investment of research efforts in this direction.

## Author Contributions

EN, MD, and ET selected, analyzed the data, and wrote the paper. NF contributed in screening literature and analyzing data.

## Conflict of Interest Statement

The authors declare that the research was conducted in the absence of any commercial or financial relationships that could be construed as a potential conflict of interest.

## Footnotes

1 http://chm.pops.int/
